# Micro-CT evaluation of rotary and reciprocating glide path and shaping systems outcomes in maxillary molar curved canals

**DOI:** 10.1007/s10266-021-00631-2

**Published:** 2021-06-25

**Authors:** Mario Alovisi, Damiano Pasqualini, Nicola Scotti, Giorgia Carpegna, Allegra Comba, Mattia Bernardi, Fabio Tutino, Mario Dioguardi, Elio Berutti

**Affiliations:** 1 Department of Surgical Sciences, Dental School, University of Turin, via Nizza, 230, 10126 Turin, Italy; 2 Department of Clinical and Experimental Medicine, University of Foggia, Foggia, Italy

**Keywords:** Shaping outcomes, Glide path, Reciprocating system, Rotary system, Micro-CT

## Abstract

The shaping outcomes after instrumentation with rotary and reciprocating glide path and shaping systems were evaluated through micro-computed tomography (Micro-CT). Thirty extracted maxillary first molars were selected. Mesio-buccal canals were randomized into two groups (*n* = 15): rotary system ProGlider and ProTaper Next X1, X2 (PG-PTN) and reciprocating system WaveOne Gold Glider and WaveOne Gold Primary (WOGG-WOG). Specimens were micro-CT scanned before, after glide path and after shaping. Increase in canal volume and surface area, percentage of removed dentin from the inner curvature, centroid shift and canal geometry variation through ratio of diameter ratios (RDR) and ratio of cross-sectional areas (RA) were measured in the apical and coronal levels and at the point of maximum curvature. The number of pecking motions needed to reach the working length (WL) was recorded. One-way ANOVA and post hoc Turkey–Kramer tests were used (*p* < 0.05). Post-glide path analysis revealed that in the coronal third, RDR was more favorable to PG and centroid shift was lower for WOGG in the apical third. Post-shaping analysis showed a reduced removal of dentin and a more favorable RA for PTN at point of maximum curvature. The number of pecking motions up to WL resulted in different between groups both for glide path and shaping phases. Despite a higher dentin removal for reciprocating instruments at the point of maximum curvature, both systems seemed to produce well-centered glide path and shaping outcomes. Rotary and reciprocating systems seemed able to respect the original canal anatomy.

## Introduction

The success of the endodontic treatment depends on an appropriate shaping and disinfection with respect to the original root canal anatomy [1, 2]. The canal scouting with stainless steel sizes 08–10 K-files provides the initial patency and the tactile feedback [3]. The subsequent glide path reduces the risk of taper lock and torsional stress of the shaping instruments [2, 4–7]. The root canal shaping optimizes disinfection and facilitates the three-dimensional obturation [2, 8, 9].

The glide path and shaping techniques require the use of stainless-steel manual or mechanical nickel-titanium (NiTi) instruments. The latter can be classified according to the type of movement performed: continuous rotation or reciprocation [10, 11]. NiTi rotary and reciprocating instruments reduce shaping time, operator fatigue and the risk of canal transportation, compared to manual ones [10–13].

ProTaper Next (PTN) rotary shaping instruments have a rectangular section and an asymmetrical rotation center which provides a "swaggering" movement and are made of a M-wire alloy. These features lead to a reduced contact between the instrument and the canal walls, a more efficient removal of debris and a greater flexibility [13–16]. Previous studies reported that ProGlider (PG) glide path single instrument seemed to improve ProTaper Next performance by positively influencing geometrical shaping outcomes and energy consumption [13, 14, 17]. Recently, WaveOne Gold (WOG) reciprocating system was introduced. These instruments have a parallelogram cross section and are manufactured by a new heat treatment that induces a Ti3Ni4 layer on instruments’ surface [18]. The reciprocating movement, the new design and alloy properties aim to improve the cyclic fatigue resistance, the root canal shaping ability and the removal of dentin debris [19, 20]. WaveOne Gold Glider (WOGG) is a single reciprocating glide path file, which was proposed in combination with WaveOne Gold. This instrument could help the operator to keep the same endodontic motor settings, optimizing ergonomics. A previous micro-CT study reported the ability of ProGlider, PathFiles and K-Files instruments to maintain canal anatomy during glide path preparation [13]. However, no comparison is available about the geometrical shaping outcomes of these different rotary and reciprocating glide path and shaping systems.

The micro-computed tomography (micro-CT) is a powerful tool for the evaluation of the shaping geometrical outcomes, allowing a non-invasive and reproducible analysis of high-resolution scans before and after treatment [21, 22].

The aim of this study is to evaluate the shaping ability of the rotary instrumentation system ProGlider and ProTaper Next compared to the reciprocating system WaveOne Gold Glider and WaveOne Gold. The null hypothesis was that the two different glide paths and shaping systems produce the same geometrical shaping outcomes.

## Materials and methods

Maxillary first permanent molars extracted for periodontal disease were selected in accordance with the local ethics committee (Protocol number CS2/1053). A sample size of 15 per group was calculated with G*Power 3.1.4 (Kiel University, Kiel, Germany) considering alpha-error = 0.05 and *ß* = 0.95. After the root debridement performed with Gracey curette 7/8 (Hu-Friedy, Chicago, IL), the specimens were dipped into a 0.01% NaOCl solution at 4°C for 24 hours and then stored in saline solution. A total of 41 teeth were selected. The specimens were mounted in the scanner with the occlusal surface against a 2 mm resin customized support fixed on a SEM stub (SkyScan 1172, Bruker micro-CT, Kontich, Belgium) to allow reproducible orientation during pre- and post-instrumentation scans [23]. Preliminary micro-CT scans were accomplished to attain a root canal anatomy outline and to ensure the respect of the inclusion criteria (SkyScan 1172, Bruker micro-CT, Kontich, Belgium). Preliminary scans were conducted with a total of 450 projections throughout a 180° rotation using a 1.0-mm-thick aluminum filter (voltage = 100 kV, current = 80 μA, source-to-object distance = 80 mm, source-to-detector distance = 220 mm, pixel binning = 8 X 8, exposure time/ projection = 0.2 s). The mesio-buccal (MB1) canals were considered only and their morphological parameters were obtained. Inclusion criteria were the following: root canal length from canal orifice to apical foramen of 12 ± 2 mm, primary canal curvature between 25°- 40° according to Schneider method on the mesio-distal plane [24], radius of curvature of 4 < r ≤ 8 mm and a point of maximum curvature located within the middle third of the root canal. Teeth with a distinct fourth canal orifice were selected to exclude samples presenting mesial roots with a single flat MB canal. Teeth with significant calcifications or not according to the inclusion criteria were excluded. The teeth were without caries, cracks and extended restorations. Of 41 teeth selected, eleven were excluded due to anatomical features. Thirty samples were randomly assigned to the two groups using a computer-generated randomization system: ProGlider and ProTaper Next rotary shaping system (group PG-PTN) (*n*=15) (Dentsply Sirona, Ballaigues, Switzerland) and WaveOne Gold Glider and WaveOne Gold reciprocating shaping system (group WOGG-WOG) (*n*=15) (Dentsply Sirona, Ballaigues, Switzerland). A single blind operator checked randomization and allocation and performed statistical analysis. One single expert operator was up-skilled on both instrumentation techniques and previously calibrated for pecking speed and pressure on the handpiece using an endodontic engine with torque measurement. A traditional access cavity preparation was designed following conventional guidelines: outline and cervical dentin were modified as needed until all orifices could be visualized in the same field of view and straight access to canal orifices could be achieved without coronal interferences [25]. Then, canal scouting was accomplished in all specimens with #10 K-file at working length (WL) using Glyde (Dentsply Sirona, Ballaigues, Switzerland) as lubricating gel (0.80 mg). WL was established with 10X magnification (OPMI Pro Ergo, Carl Zeiss, Oberkochen, Germany) when the tip was visible at the apical foramen and then subtracting 0.5 mm. In Group PG-PTN, glide path was performed with Proglider (PG) rotary single file (size 0.16, taper .02 to .082 at D16) (Dentsply Sirona, Ballaigues, Switzerland). Then, shaping was concluded with ProTaper Next (PTN) X1 (tip size 0.17 mm, taper .04) and X2 (tip size 0.25 mm, taper .06) (Dentsply Sirona, Ballaigues, Switzerland). Both PG and PTN were used with an endodontic engine X-Smart Plus (Dentsply Sirona, Ballaigues, Switzerland) with 16:1 contra angle (300 rpm, 4 Ncm) in continuous rotation up to WL. In Group WOGG-WOG, glide path was performed with WaveOne Gold Glider (WOGG) reciprocating single file (tip size 0.15, taper .017 to .085 at D16) (Dentsply Sirona, Ballaigues, Switzerland). Then, shaping was concluded with WaveOne Gold (WOG) Primary (size 0.25, taper .07) (Dentsply Sirona, Ballaigues, Switzerland). Both WOGG and WOG were used with an endodontic engine X-Smart Plus (Dentsply Sirona, Ballaigues, Switzerland) set in the “WAVEONE ALL” mode until reaching the WL. Rotary and reciprocating instruments were used with in and out motion, with no intentional brushing against canal walls. Instruments were removed from the canal and cleaned each time after three pecking motions until WL was reached. In both groups, the apical gaging was performed with K-Files to confirm the apical preparation diameter. New instruments were used for each specimen. Irrigation was completed with 5% NaOCl (Niclor 5, OGNA, Muggiò, Italy) and with 10% EDTA alternated for a total of 10 mL for each per specimen delivered with a 30-gage needle up to 4 mm from the WL. Recapitulation with a size 10 K-File was conducted between each instrument. The selected samples were scanned at high-resolution before preparation, after glide path and after shaping (100 kV, 100 μA, 16 μm resolution, Al+Cu filter and 360° rotation for a total of 2400 projections). Afterwards, the images were reconstructed with NRecon software (SkyScan 1172, Bruker micro-CT, Kontich, Belgium) using standard parameters for beam hardening and ring artifact correction and the binarized objects were analyzed with CTAn software (SkyScan 1172, Bruker micro-CT, Kontich, Belgium). Two expert operators carried out scans analysis and inter examiner agreement was calculated using weighted kappa statistics (K > 0.90). The increase in canal volume and surface area was calculated for each sample through 3D renderings. The following 2D parameters were measured starting from orthogonal cross sections: the canal centroid shift, the reduction of dentin thickness from the furcation side expressed as a percentage of the difference between pre- and post-instrumentation values, the ratio of diameter ratios (RDR) and the ratio of cross-sectional areas (RA) using ImageJ 1.43u 64-bit software (National Institute of Health, Bethesda) [13, 16]. RDR represents the instrument tendency to asymmetrically enlarge the root canal in one direction: RDR = (D/d)post/(D/d)pre, where (D/d)post is the post-preparation ratio of the major diameter (D) to the minor diameter (d) and (D/d)pre is the pre-preparation ratio of D to d. Values closer to 1 correspond to a better maintenance of the original canal geometry. RA quantifies the ability of the instrument to enlarge the root canal space: RA = Apost/Apre, where A_post_ and A_pre_ are the post-preparation and the pre-preparation cross-sectional areas, respectively. Values closer to 1 correspond to a reduced difference between post- and pre-instrumentation measurements [26]. Root sections orthogonal to the canal axis were set at 3 different levels: apical (A), 1 mm from the apical foramen; middle (M), set at the point of maximum curvature and coronal (C), set in correspondence to the middle portion of the root canal coronal third defined by 3D calculation of the root canal length from apex to orifice. These levels were selected as most representative of the critical shaping portions [27]. The bidimensional parameters were analyzed at each level except for the reduction of dentin thickness, which was evaluated only for the M level. An automated minimum threshold was set to avoid manual errors [28]. The distribution of the data was analyzed with a Shapiro–Wilk normality test. The differences of the root canal curvature at baseline were analyzed with a Kruskal–Wallis and post hoc Dunn’s tests (*P* < 0.05). One-way ANOVA and post hoc Tukey–Kramer tests were used to analyze the increase of canal surface area and volume, the centroid shift, the impact of the instrumentation on RDR and RA parameters at each level of analysis and the number of pecking motions (*P* < 0.05). All of the statistical analyses were conducted with the Minitab 15 software package (Minitab Inc., State College).

## Results

**Fig. 1:**
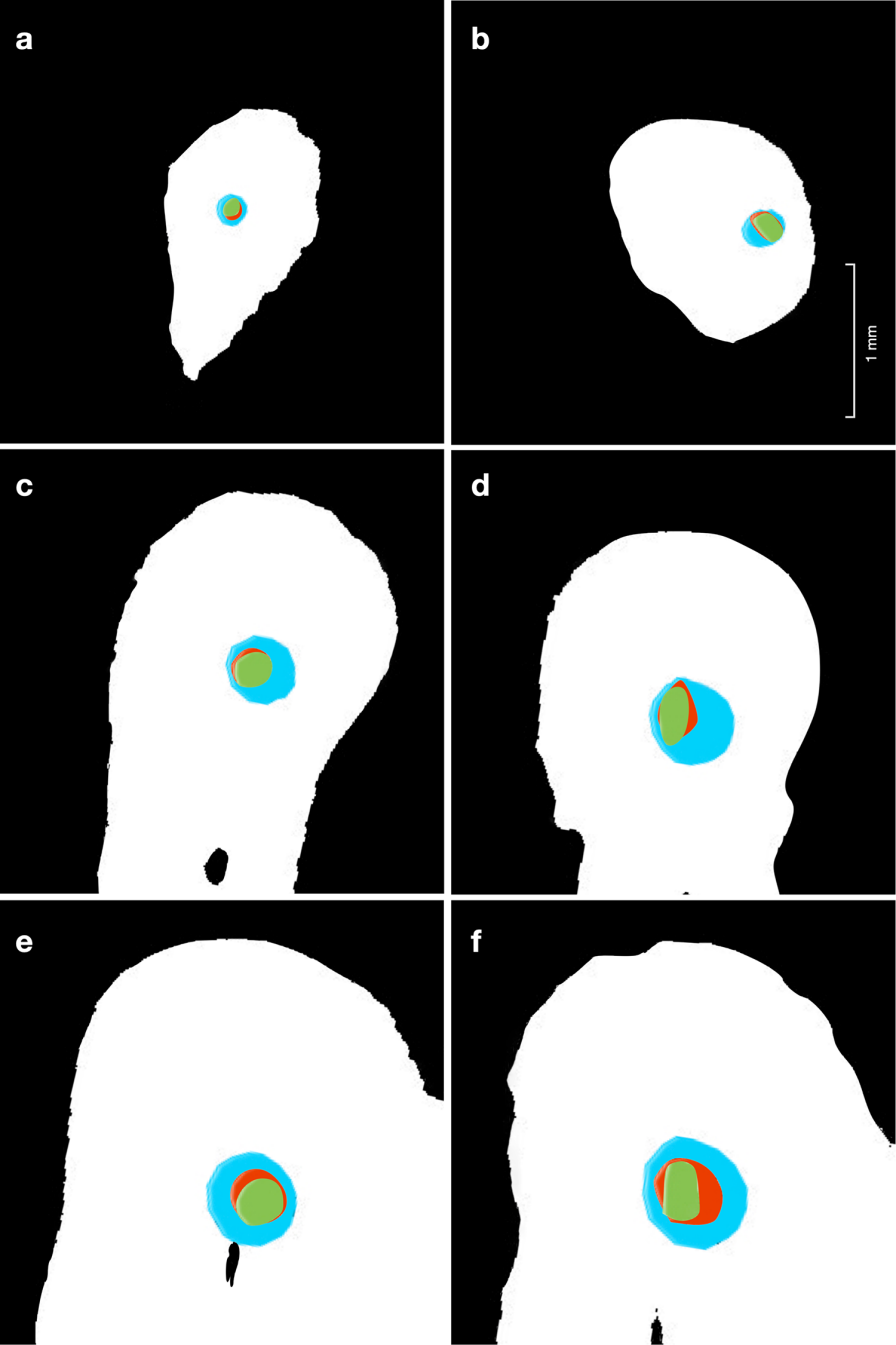
Image matching of pre-instrumentation, post-glide path and post-shaping sections according to the previously selected cutting planes. Note the difference between pre-treatment (green) post-glide path (red) and post-shaping (blue) specimens. **a)** ProGlider and ProTaper Next rotary shaping system group (PG-PTN) at the apical level of analysis (A). **b)** WaveOne Gold Glider and WaveOne Gold reciprocating shaping system group (WOGG-WOG) at A. **c)** PG-PTN at the maximum curvature level of analysis (M). **d)** WOGG-WOG at M. **e)** PG-PTN at the coronal level of analysis (C). **f)** WOGG-WOG at C

The mean canal curvature was 32.7° ± 2.9° (min = 25°, max = 38°) and 32.1° ± 3.6° (min = 26°, max = 36°) in the PG-PTN and WOGG-WOG groups, respectively, with no statistical differences (*P* = 0.21). There was no incidence of instrument fracture during canal preparation. Canal volumes, surface areas and mean apical diameters at baseline are presented in Table 1. The pre-operative values displayed homogeneity between groups (*p* > 0.05). Figure 1 represents the 2D matching of pre-operative (green), post-glide path (red) and post-shaping (blue) canal sections at the apical (A), at point of maximum curvature (M) and coronal (C) levels of analysis in all groups.

**Table 1:** Sample baseline characteristics in all groups (mean, STD). PG-PTN = ProGlider – ProTaper Next group, WOGG—WOG = WaveOne Gold Glider – WaveOne Gold group. Statistical significance indicated by *P* < 0.05. *Apical diameters (mean ± SD) at 1 mm from apical foramen

	PG-PTN	WOGG-WOG	*P*
Canal volumes (mm^3^)	1.98 ± 0.87	2.16 ± 0.77	0.19
Canal surface area (mm^2^)	14.12 ± 2.91	16.05 ± 3.35	0.13
Apical diameters* (mm)	0.16 ± 0.06	0.17 ± 0.10	0.36

Post-glide path comparisons are reported in Table 2. The mean number of pecking motions to complete glide path was 3.80 ± 1.75 in the PG group and 5.1 ± 1.90 in the WOGG group. There was a significant difference between groups (*p* = 0.02). The increase of root canal volume and surface area between groups was not significantly different (*p* > 0.05). RDR was statistically significant (*p* = 0.014) in the coronal third, showing values ​​closer to 1 in the PG group. RA value showed no differences between groups (*p* > 0.05). In the coronal and middle third, centroid shift was not significant (*p* > 0.05); while in the apical third, the difference between the two groups was significant (*p* = 0.020) with data in favor of WOGG. Finally, the percentage of dentin removed from the furcation at the point of maximum curvature showed no differences between groups (*p* > 0.05).

**Table 2:** 3D and 2D parameters utilized for post-glide path analysis in each group (PG = ProGlider; WOGG = WaveOne Gold Glider; RDR = Ratio of Diameters Ratios; RA = Ratio of Cross-Sectional Areas)

Group	Increase in canal volume (mm^3^)	Increase in canal surface area (mm^2^)		Dentinal removal from inner curvature (%)	Centroid shift (mm^−1^)	RDR (ratio)	RA (ratio)
	Mean ± SD	Mean ± SD	Level of analysis	Mean ± SD	Mean ± SD	Mean ± SD	Mean ± SD
			Coronal		0.34 ± 0.26^a^	0.94 ± 0.17^a^	1.14 ± 0.16^a^
PG	0.39 ± 0.15^a^	0.90 ± 0.21^a^	Middle	3.91 ± 2.2^a^	0.39 ± 0.30^a^	0.98 ± 0.09^a^	1.31 ± 0.07^a^
			Apical		0.37 ± 0.13^a^	0.99 ± 0.14^a^	1.15 ± 0.94^a^
			Coronal		0.38 ± 0.28^a^	0.66 ± 0.25^b^	1.47 ± 0.62^a^
WOGG	0.58 ± 0.36^a^	1.83 ± 1.44^a^	Middle	4.43 ± 2.7^a^	0.43 ± 0.25^a^	0.89 ± 0.23^a^	1.28 ± 0.28^a^
			Apical		0.25 ± 0.29^b^	1.04 ± 0.22^a^	1.16 ± 0.16^a^

Different superscript letters (^a,b^) in the same column indicate significant differences between groups (*P* < 0.05). For 2D parameters (centroid shift, RDR and RA, % dentin removal), significance was compared for the same level of analysis (coronal, middle or apical) except for the parameter % dentin removal (inner curvature), which was evaluated only for the middle (M) level

Post-shaping comparisons are reported in Table 3. The mean number of pecking motions to complete shaping was 11.6 ± 1.36 with PTN X1 and X2 and 13.9 ± 1.74 with WOG Primary. There was a significant difference between groups (*p* = 0.041). Volume and canal surface area increase were significantly different and PTN group removed less dentine compared to the WOG group (*p* = 0.003 and *p* = 0.012, respectively). In the coronal third (C), RA and RDR were not significant (*p* = 0.075 and *p* = 0.087, respectively). RDR was closer to the value of 1 for the PTN group, which had a greater tendency to work symmetrically, while RA, representing the canal widening, was close to 1 for the WOG group. At the point of maximum curvature (M), RDR was not significant (*p* = 0.056) while RA was significant and demonstrated a reduced root canal widening for the PTN group (*p* = 0.019). Apically, RDR was not significant (*p* = 0.094), and RA showed values ​​closer to 1 for the WOG group (*p* = 0.062). Between the two groups, there were no statistically significant differences about the displacement of the centroid in any of the three levels of analysis (*p* > 0.05). Finally, the percentage of dentin removed from the furcation at the point of maximum root curvature had an average of 11.20% for the PTN system and 19.61% for the WOG system, this difference being statistically significant (*p* = 0.016).

**Table 3:** 3D and 2D parameters utilized for post-shaping analysis in each group (PTN = ProTaper Next; WOG = WaveOne Gold; RDR = Ratio of Diameters Ratios; RA = Ratio of Cross-Sectional Areas)

	Increase in canal volume (mm^3^)		Increase in canal surface area (mm^2^)			Dentinal removal from inner curvature (%)		Centroid shift (mm^−1^)		RDR (ratio)		RA (ratio)	
Group		Mean ± SD		Mean ± SD	Level of analysis		Mean ± SD		Mean ± SD		Mean ± SD		Mean ± SD
					Coronal				0.61 ± 0.36^a^		0.85 ± 0.27^a^		2.80 ± 0.50^a^
**PTN**		0.87 ± 0.50^a^		2.77 ± 2.04^a^	Middle		11.20 ± 10.39^a^		0.73 ± 0.26^a^		0.95 ± 0.24^a^		1.93 ± 0.62^a^
					Apical				0.45 ± 0.29^a^		0.90 ± 0.17^a^		1.61 ± 0.34^a^
					Coronal				1.03 ± 0.37^a^		0.49 ± 0.21^a^		1.75 ± 1.35^a^
**WOG**		2.14 ± 1.16^b^		4.79 ± 3.44^b^	Middle		19.61 ± 9.62^b^		1.14 ± 0.41^a^		0.66 ± 0.17^a^		3.31 ± 1.35^b^
					Apical				0.58 ± 0.45^a^		0.96 ± 0.39^a^		1.19 ± 1.17^a^

Different superscript letters (^a,b^) in the same column indicate significant differences between groups (*P* < 0.05). For 2D parameters (centroid shift, RDR and RA, % dentin removal), significance was compared for the same level of analysis (coronal, middle or apical) except for the parameter % dentin removal (inner curvature), which was evaluated only for the middle (M) level

## Discussion

In this study, both tested rotary and reciprocating glide path and shaping systems produced a well-centered preparation respecting the original canal anatomy. However, the null hypothesis was partially rejected, and some significant differences of the geometrical parameters were observed. These differences could be considered helpful for the clinical selection of the right shaping system.

All tested instruments had similar tip size, to compare the different shaping systems’ outcomes through micro-CT analysis. The micro-CT analysis of the post-operative variations is an effective indicator of the instruments’ shaping ability [15, 21–23, 29, 30]. An extracted tooth model is usually well transferable to the clinical situation [31], but the homogeneity of pre-operative sample characteristics is essential to ensure an adequate standardization [13]. Teeth with a single flat mesio-buccal canal were excluded and rounded MB1 separated canals were preferred to improve standardization and to optimize the micro-CT analysis [13]. In the present study, baseline homogeneity was assumed between groups for root canal volume, surface area and apical diameters (Table 1). The results were coherent with data in literature and appeared adequate for the shaping systems used [32]. Both rotary and reciprocating instruments considered in this study are recommended to be utilized with a brushing motion on the outstroke to eliminate coronal interferences [33, 34]. However, in the present study, an intentional brushing motion was avoided to standardize the operator’s shaping movements for each technique [13]. Gel chelating agents were used for canal scouting, while 10% EDTA liquid solution and 5% NaOCl were alternated during glide path and shaping. This irrigation protocol was selected to maintain the same experimental conditions of a previous study [13]. Whitbeck et al. reported that higher transportation and increased canal volume were observed in samples irrigated with EDTA 17% solution and scanned with micro-CT [35]. However, the effects of lower EDTA concentrations on shaping outcomes are still unclear; however, this may represent a limit of this study and should be further investigated.

Glide path and preliminary coronal enlargement simplify the use of the NiTi shaping files improving their performance and respect of the original anatomy [14, 17, 36]. Therefore, the study of the root canal geometrical parameters after glide path could be an indicator for the subsequent shaping outcomes [17]. Moreover, this instrumentation phase may be associated with higher rate of procedural errors, blocks and ledges [27]. Thus, several studies reported the benefits of the mechanical glide path, in terms of simplicity, time required for shaping and maintenance of the canal anatomy [6, 26].

In the present study, the post-glide path micro-CT tridimensional parameters revealed that WOGG demonstrated an aptitude to remove more dentine in the root canal coronal and middle third, accordingly with its higher conical shape compared with PG, even if the results were not statistically significant. This aspect could be related to the necessity to hold down the number of pecking motions necessary to reach the working length with a single reciprocating shaping instrument [20]. These findings seem partially in contrast with a previous study, probably due to the anatomy and the degree of curvature of the tested roots [37].

Regarding the evaluated bidimensional parameters, post-glide path analysis showed that in the coronal third PG, instrument seemed to facilitate a better symmetrical shaping and a lower tendency to canal transportation with RDR value closer to one. This result may due to the geometrical features of PG, which has a lower conical shape in the coronal and medium third. A previous study confirmed the ability of PG to create a symmetrical glide path and initial coronal flaring, due to its high flexibility [13]. Nevertheless, both PG and WOGG instruments showed a tendency to enlarge the coronal and middle root canal portions due to their progressive tapered design. At the apical level of analysis, WOGG remained more centered, probably due to its specific reciprocating movement and the lower apical diameter. Previous studies correlated the reciprocating motion to a more centered preparation compared with continuous rotating movement, especially in the apical third [38]. However, these results seem in contrast with a previous study, probably due to the different root cross sections levels analyzed [37].

Collected tridimensional data from post-shaping analysis identified a statistically significant difference between PTN and WOG group with respect to the variation in canal volume and surface area, with a higher increment in the WOG group. Root canal changes after shaping are affected by different factors, such as root canal anatomy, file design, alloys and instrumentation sequence [2, 8, 21]. The PTN off-centered cross section gives the file a reduced pattern of contact between the instrument and the canal walls enhancing flexibility and debris removal [16]. Moreover, the asymmetric rotary motion of the ProTaper Next system leads to the same preparation size with smaller and more flexible instruments [39]. Therefore, in this study, PTN instrumentation sequence provided a lower number of pecking motions necessary to reach the WL. On the other hand, the reciprocating movement was correlated to a lower straightening of the canal curvature and the Gold heat-treated instruments demonstrated enhanced flexibility compared with conventional NiTi and M-Wire instruments [40, 41]. However, in the present study, the dentinal removal on the furcation side at the point of maximum curvature resulted more accentuated for the WOGG-WOG system, probably due to the significantly higher number of pecking motions required to reach the WL. Root canal transportation is an aberration that may occur during shaping implying an excessive dentin removal [2, 3]. The canal curvature straightening leads to a reduction of the dentin wall thickness and may negatively influence the long-term prognosis of the tooth [2, 3, 14, 27].

Bidimensional post-shaping analysis demonstrated that in the coronal third, WOG tended to create a reduced widening of the root canal and the ratio between the post- and pre-instrumentation areas (RA) was barely significant. These data could be easily understood by comparing the geometry and the different taper variations between the groups: the WOG Primary shows a 3% taper at 13 mm from the tip, while PTN X2 has a 6% taper. However, post-shaping RA values were statistically significant in the medium third, where PTN systems resulted more preservative in correspondence of the maximum curvature. PTN X2 is smaller than the envelope of motion it creates, thereby being more flexible and sensitive to the curvatures [42]. Moreover, glide path with ProGlider could reduce the stress stored by the ProTaper Next X1 during shaping positively influencing the centering ability of ProTaper Next X2 [17, 42]. These results seem in accordance with existing study which showed a slightly more accentuated transportation tendency of the WOG system at the midroot level [43]. In the apical third, the reciprocating movement seemed to allow a more conservative preparation. and RA value was significantly lower in the WOG group. This effect may be correlated to the theory of the balanced forces exerted by the instruments during shaping [3, 44].

## Conclusion

Within the limits of this study, the WOGG-WOG shaping system seemed to promote a centered root canal instrumentation, especially in the apical third, with a higher dentinal removal at the point of maximum curvature, compared with the PG-PTN rotary system. The greater volume and canal surface increase obtained with the reciprocating system could be related to the higher number of pecking motions needed to complete shaping.
